# MicroRNA hsa-miR-150-5p inhibits nasopharyngeal carcinogenesis by suppressing PYCR1 (pyrroline-5-carboxylate reductase 1)

**DOI:** 10.1080/21655979.2021.1995102

**Published:** 2021-12-02

**Authors:** Zhiqun Li, Xiaoliu Zhou, Jiajun Huang, Zhencai Xu, Chengliang Xing, Junwei Yang, Xuejun Zhou

**Affiliations:** Department of Otolaryngology Head and Neck Surgery, The First Affiliated Hospital of Hainan Medical University, Haikou, China

**Keywords:** Hsa-miR-150-5p, PYCR1, nasopharyngeal cancer

## Abstract

Nasopharyngeal cancer is a rare cancer type, but with a low five-year survival rate. Dysregulation of pyrroline-5-carboxylate reductase 1 (PYCR1) and microRNA hsa-miR-150-5p is involved in the development of various cancers. However, the molecular mechanism of the hsa-miR-150-5p-PYCR1 axis in nasopharyngeal cancer remains unclear. To identify the mechanism of the hsa-miR-150-5p-PYCR1 axis, the expression of hsa-miR-150-5p and PYCR1 in nasopharyngeal cancer tissues and cells was first measured by reverse transcription quantitative polymerase chain reaction. The luciferase and RNA pull-down assays were used to confirm the interaction between hsa-miR-150-5p and PYCR1. The overexpression of hsa-miR-150-5p and PYCR1 was detected by cell viability, proliferation, western blotting, migration, and invasion in nasopharyngeal cancer cells. The expression levels of hsa-miR-150-5p was reduced in the nasopharyngeal cancer tissues and cells and were negatively correlated with the PYCR1 levels. The upregulation of hsa-miR-150-5p significantly repressed cell growth and promoted apoptosis. However, the upregulation of *PYCR1* expression significantly promoted nasopharyngeal carcinogenesis, which could abolish the inhibitory effect of hsa-miR-150-5p. In conclusion, we clarified that hsa-miR-150-5p attenuated nasopharyngeal carcinogenesis by reducing the *PYCR1* expression levels. This provides a new perspective of nasopharyngeal cancer involving both hsa-miR-150-5p and PYCR1 for the treatment of nasopharyngeal cancer.

## Introduction

Nasopharyngeal cancer (NPC) is highly prevalent in northern Africa and south-eastern Asia, especially in southeastern China [1]. Radiotherapy, chemotherapy, or combination treatments are common strategies that are used to treat patients with NPC. Unfortunately, a low five-year survival rate remains a serious problem [2]. Many studies have suggested the dysregulation of cell growth and apoptosis of NPC cells [3,4]; however, the potential mechanism of the pathogenesis of NPC needs to be elucidated.

MicroRNAs (miRNAs) are a variety of non-coding RNAs consisting of 19–24 nucleotides, that regulate cellular biological processes by binding to the target gene 3 untranslated regions (UTRs) [5]. A growing body of evidence indicates that these miRNAs play key roles in carcinogenesis, namely cell growth, differentiation, and apoptosis [6,7]. Hsa-miR-150-5p participates in the regulation of various cancers, including breast cancer, colorectal cancer, and oral squamous cell carcinoma (OSCC) [8–10]. For instance, hsa-miR-150-5p expression was dramatically decreased in OSCC tissues, which further repressed cell growth and enhanced apoptosis of OSCC cells [10]. Additionally, hsa-miR-150-5p expression is downregulated in breast cancer tissues and attenuates the proliferation and migration of breast cancer cells [8]. Notably, hsa-miR-150-5p has also been associated with NPC development [11–14]. Studies have reported that hsa-miR-150-5p hampers the NPC cell migration by reducing CD44 molecule (CD44) expression [11]. High levels of hsa-miR-150-5p inhibiting glycogen synthase kinase 3 beta (GSK3β) protein levels have contributed to poor prognosis in NPC patients [12]. In addition, Zhu et al. [14] found that hsa-miR-150-5p expression was significantly decreased in NPC, and its knockdown promoted the proliferation, migration, and invasion of NPC cells. However, whether hsa-miR-150-5p plays a role in *PYCR1* expression (pyrroline-5-carboxylate reductase 1) remains unclear.

*PYCR1* encodes an enzyme that catalyzes the last step of proline synthesis, which plays a key role as an oncogene in cancer progression [15–17]. For instance, the downregulation of PYCR1 inhibited the growth of lung adenocarcinoma cells by repressing the Janus kinase (JAK)-signal transducer and activator of transcription-3 (STAT3) signaling pathway [15]. Wang et al. reported that PYCR1 enhanced non-small-cell lung cancer (NSCC) cell growth when miR-488 was downregulated [16]. Another study also indicated that the downregulation of PYCR1 significantly suppressed prostate cancer cell growth and colony formation, but enhanced cell apoptosis [17]. However, the effect of PYCR1 on NPC cells remains unknown.

In this study, we sought to elucidate the regulatory mechanism of hsa-miR-150-5p-PYCR1 in NPC. We hypothesized that hsa-miR-150-5p might hamper cell growth during NPC development by repressing *PYCR1* expression. Our study may provide a good potential therapeutic approach for the clinical treatment of patients with NPC.

## Methods

### Tumor specimens and cell lines

Tumor tissues and adjacent normal tissues (>5 cm away from the tumor margin) were collected from patients with NPC (n = 38) between January 2020 and January 2021 from our hospital with informed consent. The clinical baseline characteristics of the patients with NPC are shown in Supplementary Table S1. Informed consent was obtained from all individuals, and the research protocol was approved by the Ethics Committee of The First Affiliated Hospital of Hainan Medical University (Approval no. KY2019012). Normal human nasopharyngeal epithelial (NP69) and NPC cells (TW03, C666-1, and SUNE-1) were obtained from the American Type Culture Collection (Manassas, VA, USA). Cells were maintained in RPMI-1640 medium (Gibco, USA) containing 10% fetal bovine serum (FBS) (Gibco, USA) in a cell incubator at 37°C and 5% CO_2_.

### Reverse transcription quantitative polymerase chain reaction (RT-qPCR)

The total RNA from the tissues and cells was extracted using Trizol (Invitrogen, USA) and was reverse transcribed using a cDNA Reverse Transcription Kit (Liankebio, China). SYBR Premix Ex Taq (Takara, Japan) was used to detect *PYCR1* expression. Tissue and cell miRNAs were extracted using an miRNA Isolation Kit (Thermo Fisher, USA) and reverse transcribed using the TaqMan MicroRNA Reverse Transcription Kit (Thermo Fisher, USA). The TaqMan OpenArray Real-Time PCR Master Mix (Thermo Fisher, USA) was used to detect hsa-miR-150-5p expression. The expression of *PYCR1* and hsa-miR-150-5p was normalized to glyceraldehyde-3-phosphate dehydrogenase (GAPDH) and Uracil6 (U6), respectively, using the 2-^ΔΔCt^ method [18]. The primers used are listed in Table 1.

**Table 1.** Sequence of PCR primers used in this study

Gene name	Primer type	Sequence
miR-150-5p	Forward	5ʹ-TCGGCGTCTCCCAACCCTTGTAC-3ʹ
	Reverse	5ʹ-GTCGTATCCAGTGCAGGGTCCGAGGT-3ʹ
CSE1L	Forward	5ʹ-TGACCAA-CACTCCAGTCGTG-3’
	Reverse	5ʹ-GTCCAGCTTCACCTTGTCCA-3’
GAPDH	Forward	5ʹ-GGAGCGAGATCCCTCCAAAAT-3’
	Reverse	5ʹ-GGCT-GTTGTCATACTTCTCATGG-3’
U6	Forward	5ʹ-CTCGCTTCGGCAGCACATATACT-3ʹ
	Reverse	5ʹ-ACGCTTCACGAATTTGCGTGTC-3ʹ

### Cell transfection

All transfectants, including *PYCR1* overexpression plasmid and empty vector, hsa-miR-150-5p mimic, and mimic negative control (NC) were purchased from Thermo Fisher (USA). Before subsequent functional experiments, C666-1 and SUNE-1 cells were transfected with the Lipo3000 (Invitrogen, USA) for 48 h.

### Cell counting kit-8 (CCK-8) detection

The transfected C666-1 and SUNE-1 cells (5 × 10^3^) were seeded into 96-well plates and examined using the CCK-8 kit (Cat#: K1018; APExBIO, China) according to the previous study [19]. At 0, 24, 48, and 72 h, 10 µL CCK-8 buffer was added to the plates and incubated for another 2 h. Finally, the optical density at 450 nm (OD_450_) was obtained using a multimode plate reader (Thermo Fisher, USA).

### 5ʹ-Bromo-2ʹ-deoxyuridine (BrdU) detection

The transfected C666-1 and SUNE-1 cells (2 × 10^4^) were seeded into 96-well plates. At 70% confluence, the cells were washed twice, and BrdU (Cat#: 6813, CST, USA) labeling solution was added to the cells. After 6 h of incubation, the cells were washed twice, fixed, and denatured by the fixation/denaturation solution. The BrdU antibody was then added and the cells were incubated for 2 h. Next, the cells were washed twice, and the secondary horse radish peroxidase (HRP)-mouse antibody was used for another 2 h of incubation. Finally, the cells were washed twice and HRP substrate TMB was added to the cells, and the OD_450_ was obtained on a multimode-plate-reader (Thermo Fisher, USA) [20].

### Wound healing detection

The transfected C666-1 and SUNE-1 cells (1 × 10^6^) were seeded into 6-well plates. After the cells reached 70% confluence, the diameter through the plate was drawn using a sterile pipette, and the floating cells were washed away. At 0 and 24 h, images of the scratches were obtained using a light microscope and recorded [21].

### Cell invasion detection

Matrigel (Corning, USA) was added into the transwell chamber with 8-µm pore (Cat#: #3244, Coring, USA). RPMI-1640 medium containing 10% FBS was added to the lower chambers. The transfected C666-1 and SUNE-1 cells were seeded into the upper chamber. After 48 h, the invaded cells were fixed with 4% paraformaldehyde, washed twice with PBS, and stained with 0.1% crystal violet. Subsequently, the invaded cells were imaged under a microscope (Olympus, Japan) [22].

### Luciferase detection

The pmiRGLO plasmid containing PYCR1 3ʹ-UTRs wild-type (WT) or mutant (MUT) sequences was provided by Tuoran Co., Ltd (Shanghai, China). C666-1 and SUNE-1 cells (5 × 10^5^) were seeded into 24-well plates, and the cells were transfected with the plasmids, and either miR-NC or hsa-miR-150-5p using Lipo3000. After 48 h, firefly and Renilla luciferase activities were examined using the Luciferase Assay Kit (RG027, Beyotime, China). Renilla luciferase activity was used as an internal control [23].

### RNA-pull down analysis

Biotin-labeled miR-185-5p (Bio-miR-185-5p) and miR-185-5p negative control (Bio-NC),, were obtained from Thermo Fisher (USA). The cell lysate suspension was mixed with Bio-hsa-miR-150-5p or Bio-NC and incubated with streptavidin beads (Cat#: #88,817, Thermo Fisher, USA) at 4°C overnight. Subsequently, the eluted solution was collected and purified using a kit (DP412, Tiangen, China). PYCR1 expression was detected by RT-qPCR [24].

### RNA immunoprecipitation (RIP) assay

The EZ-Magna RIP Kit (Millipore, Bedford, MA, USA) was used for the RIP assay, as per the manufacturer’s protocol. In brief, C666-1 and SUNE-1 cells were lysed with RIP lysis buffer. The cell extracts were then co-incubated with RIP buffer containing magnetic beads coupled to a human anti-Ago2 antibody (Millipore) or IgG, and then mixed with protease K to digest proteins. The immunoprecipitated RNA was isolated, purified, and analyzed using western blotting [25].

### Western blot analysis

Proteins from the transfected C666-1 and SUNE-1 cells were obtained using radioimmunoprecipitation assay (RIPA) buffer (P0013 C, Beyotime, China). The 20 µg per well protein sample was loaded onto 10% sulfate-polyacrylamide gel electrophoresis (SDS-PAGE) and transferred to polyvinylidene fluoride (PVDF) membranes. After blocking with 5% bovine serum albumin, anti- PYCR1 (Cat#: 47935S, CST, USA), anti-Bax (Cat#: ab32503, Abcam, UK), Anti- Bcl-2 (Cat#: ab32124, Abcam, UK), and anti-GAPDH (Cat#: 5714, CST, USA) diluted 1:1,000 were used to incubate the membranes overnight at 4°C. Subsequently, anti-HRP-Rabbit (Cat#: 7074, CST, USA) was used to incubate the membranes for 1 h. Electrogenerated chemiluminescence (ECL) reagents (P0018S, Beyotime, China) were used to develop the protein bands. GAPDH protein level was used as an internal control [26].

### Statistical analysis

The paired *t*-test, one-way analysis of variance (ANOVA), and two-way ANOVA were used to compare two groups and multiple groups, respectively, using GraphPad 8.0 (GraphPad, USA). The correlation between hsa-miR-150-5p and PYCR1 in NPC tissues was analyzed using Spearman’s correlation analysis. Data are shown as mean ±standard deviation (SD) from triplicate experiments. Statistical significance was set at *P* < 0.05.

## Results

In this study, we aimed to explore the role of hsa-miR-150-5p-PYCR1 in NPC. We conducted a series of in vitro experiments and found that hsa-miR-150-5p inhibited the activity, proliferation, migration, and invasion, as well as promoted apoptosis of NPC by negatively regulating PYCR1. Our data are the first to investigate the function and clinical expression of hsa-miR-150-5p-PYCR1 in NPC, providing new insights into the pathogenesis of NPC.

### PYCR1 and hsa-miR-150-5p were identified to be potential participants in NPC

**Figure 1.**
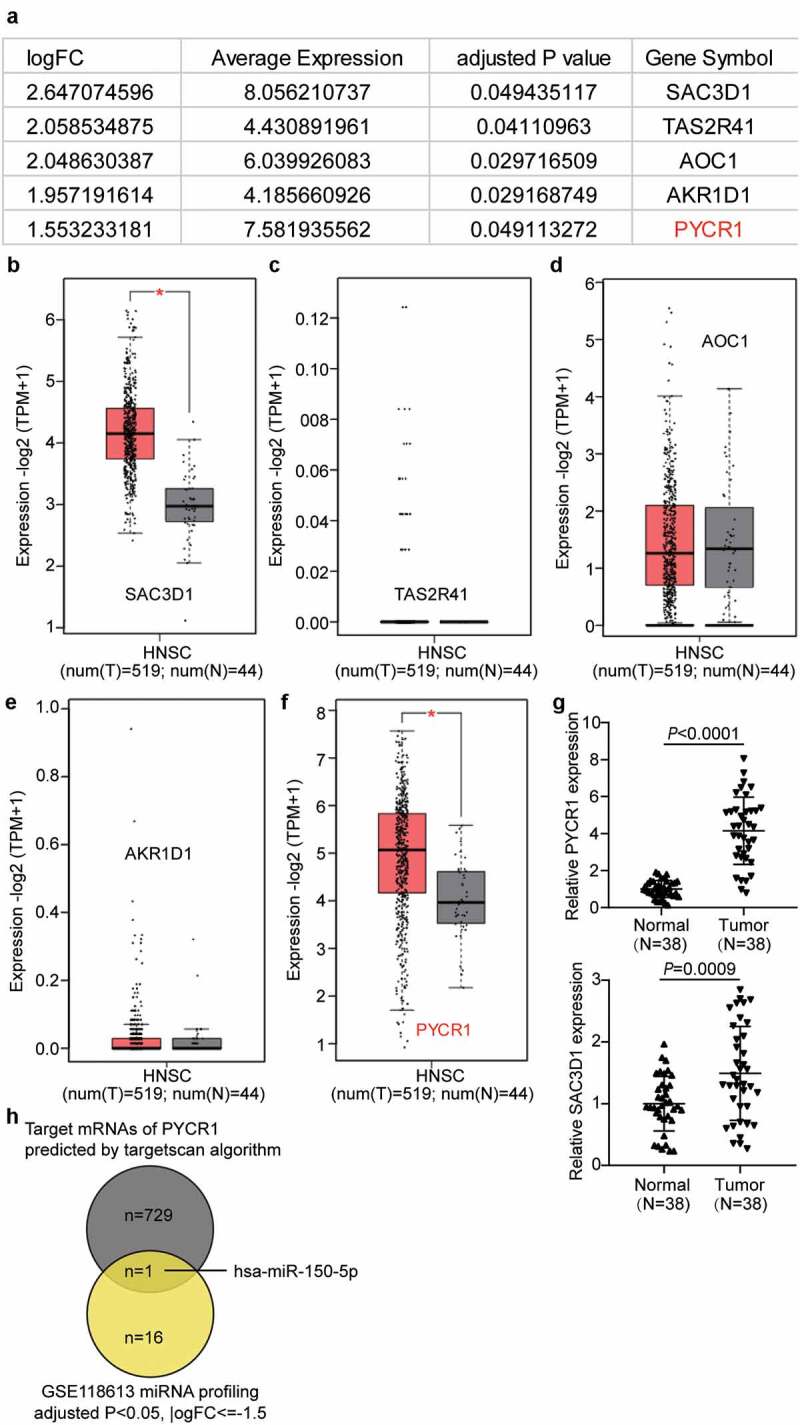
PYCR1 and hsa-miR-150-5p were selected to be studied in this research

We first analyzed the mRNA profiling of GSE64634, and the results showed that five genes were conspicuously increased in NPC with the criteria of adjusted P < 0.05, and logFC ≥1.5 (Figure 1a). We investigated the GEPIA database for expression in head and neck squamous cell carcinoma. Among the five genes, *SAC3D1* and *PYCR1* were also significantly upregulated in the GEPIA HNSC data (Figure 1b–f). Therefore, the expression of *SAC3D1* and *PYCR1* in clinical tissues was detected by RT-qPCR. RT-qPCR showed that both SAC3D1 and PYCR1 levels were increased in NPC tissues, but PYCR1 was upregulated (Figure 1g). In addition, we noticed that PYCR1 has been reported to be a significant cancer driver and its silencing could significantly suppress carcinogenesis and progression [15–17,27–29]. Nonetheless, the functions of PYCR1 in nasopharyngeal cancer have not been studied. We speculated that PYCR1 could be a cancer driver in NPC. Thus, we selected PYCR1 as the study object. To identify an upstream miRNA regulator of PYCR1 in NPC, we analyzed the miRNA profiling of GSE118613, and the results showed that 17 miRNAs were significantly downregulated in NPC with adj.P < 0.05, and logFC<-1.5 (Supplementary Table S1). By intersecting the 17- miRNA list with the predicted target miRNAs of PYCR1 by Targetscan (Supplementary Table 2), hsa-miR-150-5p was identified (Figure 1h). It has been reported that hsa-miR-150-5p could be a potent carcinogen inhibitor in NPC [13]. Thus, we aimed to study the biological roles of hsa-miR-150-5p and PYCR1 in NPC cell phenotypes.

(a) The significantly upregulated genes in NPC in GSE64634 data. Five genes were identified. Selection criteria: adjusted P < 0.05, logFC≥1.5. (b–f) The expression of the five genes in GEPIA HNSC data. HNSC: head and neck squamous cell carcinoma. (g) RT-qPCR analysis of SAC3D1 and PYCR1 expression in NPC tissues and normal tissues. n = 38. **, *P* < 0.001. (h) The candidate miRNA that both targets PYCR1 mRNA and significantly downregulates in NPC (data from GSE118613 data analysis).

### Hsa-miR-150-5p suppressed NPC cell progression

**Figure 2.**
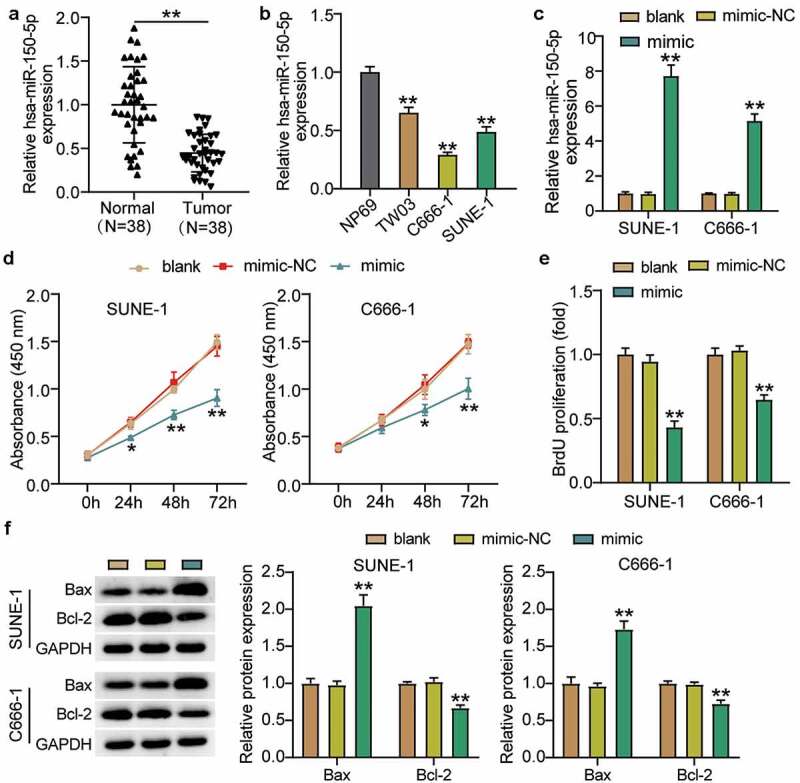
Hsa-miR-150-5p inhibited NPC cells viability and proliferation

**Figure 3.**
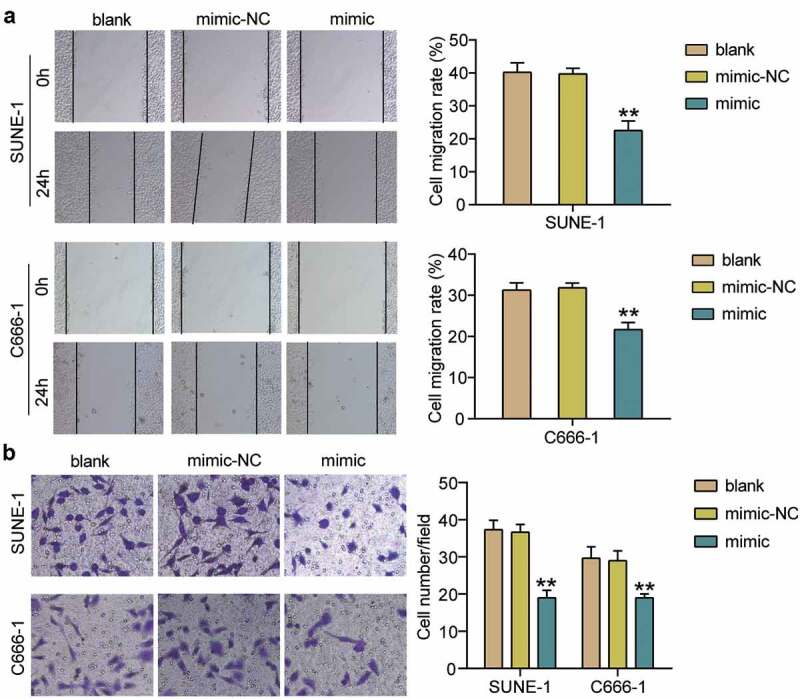
Hsa-miR-150-5p inhibited cell migration and invasion of NPC cells

To clarify the effect of hsa-miR-150-5p in NPC, we analyzed NPC tissues and hsa-miR-150-5p expression. We found that hsa-miR-150-5p expression was significantly repressed by 50% in the NPC tissue samples, and the TW03, C666-1, and SUNE-1 NPC cells showed a notable decrease in the hsa-miR-150-5p expression levels compared with normal cells (Figures 2a,b). We performed further studies in C666-1 and SUNE-1 cell lines as these indicated the lowest hsa-miR-150-5p expression in the cells. Next, we transfected hsa-miR-150-5p mimics and NC into the C666-1 and SUNE-1 cells. The results illustrated that the mimic groups in the C666-1 and SUNE-1 cells were enhanced by approximately an 8-fold and 5-fold hsa-miR-150-5p expression compared with blank cells, respectively (Figure 2c). Additionally, the mimic groups conspicuously attenuated cell viability compared with the blank cells in both cell lines (Figure 2d). Furthermore, the mimics groups decreased cell proliferation by approximately 50% compared with blank cells in both cell lines (Figure 2e). In addition, western blotting showed that hsa-miR-150-5p overexpression inhibited the level of BCL-2 protein and upregulated BAX (Figure 2f). Meanwhile, the mimic groups in C666-1 and SUNE-1 cells showed 50% and 30% cell migration compared with blank cells, respectively (Figure 3a). Moreover, the mimic groups in C666-1 and SUNE-1 cells showed approximately 50% and 30% decrease in the cell invasion compared with blank cells, respectively (Figure 3b). The data showed that hsa-miR-150-5p attenuated the progression in NPC.

(a)RT-qPCR detection of hsa-miR-150-5p expression in NPC tissues and normal tissues. n = 38. **, *P* < 0.001. (b) RT-qPCR detection of hsa-miR-150-5p expression in NPC cells lines (TW03, C666-1 and SUNE-1) and normal cell (NP69). **, *P* < 0.001 compared with NP69. (c) Measurement of hsa-miR-150-5p expression in C666-1 and SUNE-1 cells transfected with mimic-NC and hsa-miR-150-5p mimic by RT-qPCR. (d) Cell viability was detected in C666-1 and SUNE-1 cells transfected with mimic-NC and hsa-miR-150-5p mimic by CCK-8 assay. (e) Cell proliferation was detected in C666-1 and SUNE-1 cells transfected with mimic-NC and hsa-miR-150-5p mimic. (f) Bax and Bcl-2 protein expression was detected in C666-1 and SUNE-1 cells transfected with e mimic-NC and hsa-miR-150-5p mimic by western blot assay. n = 3. *, *P* < 0.05; **, *P* < 0.001 compared with blank. NC, negative control; mimic, hsa-miR-150-5p mimic.

(a) Cell migration was detected in C666-1 and SUNE-1 cells transfected with mimic-NC and hsa-miR-150-5p mimic. (b) Cell invasion was detected in C666-1 and SUNE-1 cells transfected with mimic-NC and hsa-miR-150-5p mimic. n = 3. **, *P* < 0.001 compared with blank. NC, negative control; mimic, hsa-miR-150-5p mimic.

### PYCR1 is a target of hsa-miR-150-5p

**Figure 4.**
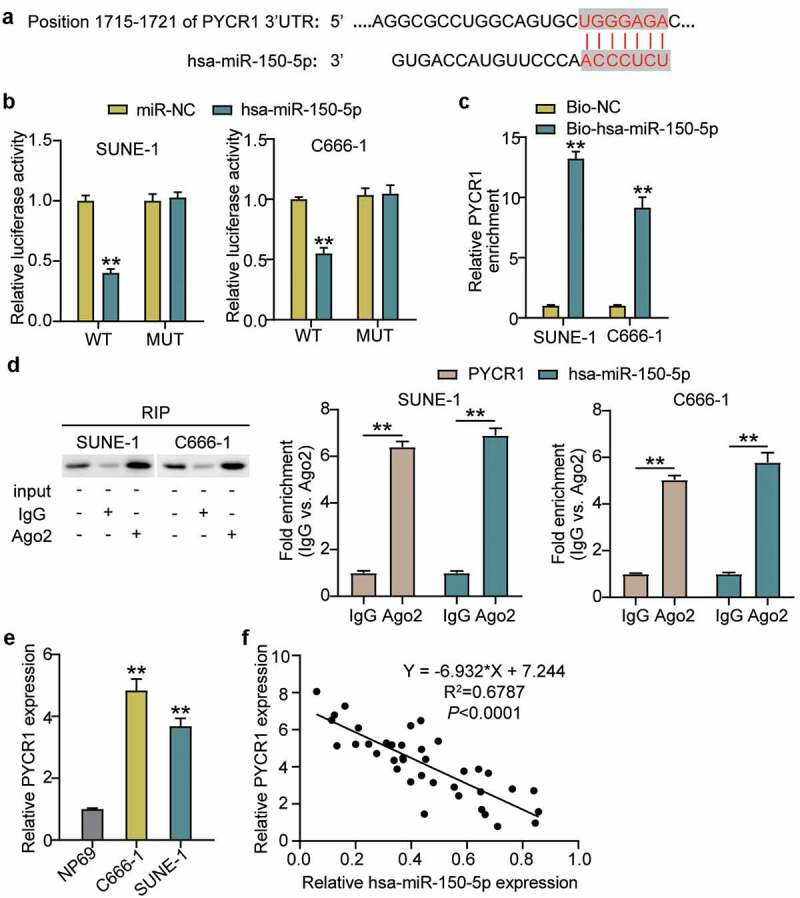
PYCR1 was a target of hsa-miR-150-5p

The binding sequence between PYCR1 and hsa-miR-150-5p is shown in Figure 4a using TargetScan Human 7.2. Next, we transfected plasmids containing PYCR1 3-UTR WT or MUT sequences, and hsa-miR-150-5p-NC or hsa-miR-150-5p-mimic into C666-1 and SUNE-1 cells. The results showed that the luciferase activities of WT +mimic groups were dramatically downregulated by approximately 50%, while the MUT +mimic groups showed no difference in both cells (Figure 4b), which was further verified by RNA-pull down assay (Figure 4c). To further confirm the interaction between PYCR1 and hsa-miR-150-5p, an RIP assay was performed. A prominent enrichment of hsa-miR-150-5p and PYCR1 was observed in Ago2 (Figure 4d), suggesting that the specificity of the interaction between hsa-miR-150-5p and PYCR1. The C666-1 and SUNE-1 cells showed significantly enhanced *PYCR1* expression compared with normal cells (Figure 4e). Additionally, hsa-miR-150-5p was negatively correlated with PYCR1 levels in NPC tumor tissues (Figure 4f). Taken together, these data suggest that hsa-miR-150-5p directly targets PYCR1.

(a) Bioinformatics analysis showed the predicted binding sequence of PYCR1 3ʹ-UTR. (b) Dual luciferase assay was performed in cells co-transfected with plasmids PYCR1-WT or PYCR1-MUT and miR-NC or hsa-miR-150-5p mimic in C666-1 and SUNE-1 cells. **, *P* < 0.001 compared with miR-NC. (c) RNA pull-down analysis of PYCR1 expression in C666-1 and SUNE-1 cells. **, *P* < 0.001 compared with Bio-NC. (d) Association of hsa-miR-150-5p and PYCR1 expression levels with AGO2 were determined by RIP-western blotting with AGO2 antibody and RT-qPCR used for RIP analysis as compared with IgG group. **, *P* < 0.001. (e) RT-qPCR detection of expression of PYCR1 in the NPC C666-1 and SUNE-1 cells and NP69cells. **, *P* < 0.001 compared with NP69. (f) Correlation analysis between the hsa-miR-150-5p expression and expression in the NPC tumor tissues. n = 3. WT, wild-type; MUT, mutant; NC, negative control.

### Hsa-miR-150-5p attenuated the development of NPC via repressing PYCR1

**Figure 5.**
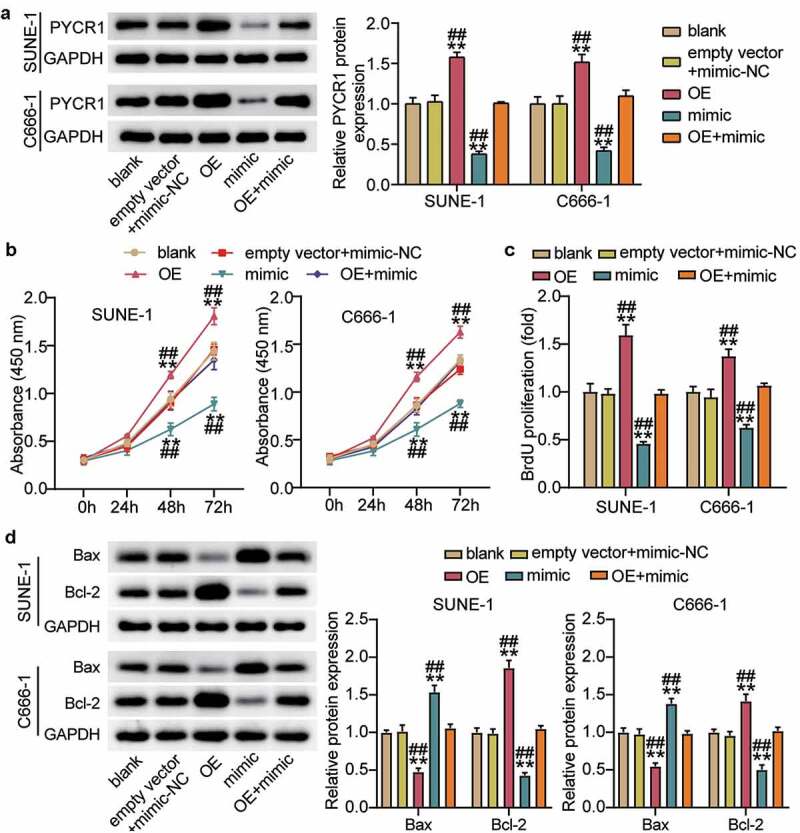
Hsa-miR-150-5p targeting PYCR1 repressed cell proliferation, but enhanced cell apoptosis of NPC cells

**Figure 6.**
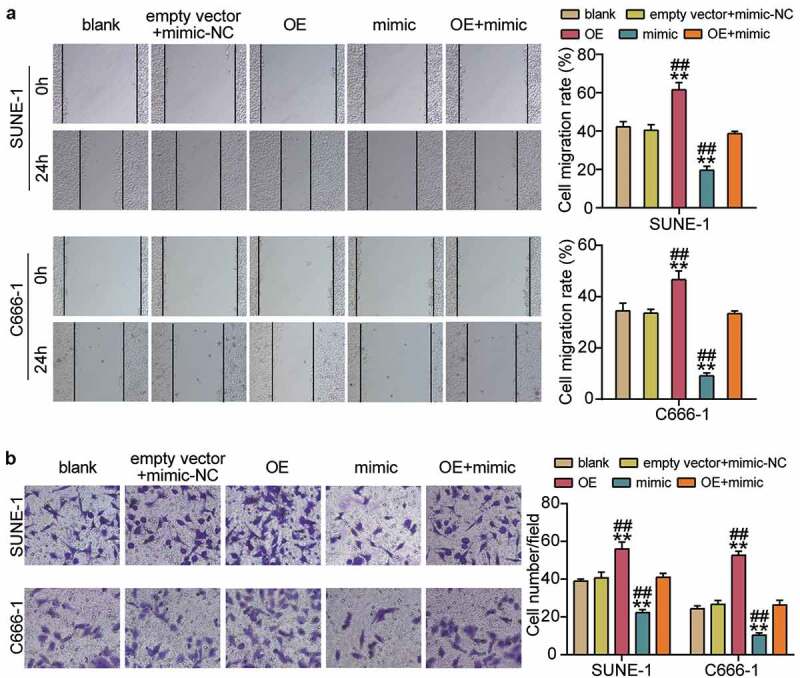
Hsa-miR-150-5p targeting PYCR1 suppressed cell migration and invasion of NPC cells

To corroborate the role of the hsa-miR-150-5p-PYCR1 axis in NPC, we transfected empty vector+mimic-NC, PYCR1 overexpression (OE), hsa-miR-150-5p mimic, and OE+ mimic into C666-1 and SUNE-1 cells. The results showed a nearly 1.5-fold increase in PYCR1 protein level in the OE groups, while a 50% decrease in PYCR1 protein expression was observed in both C666-1 and SUNE-1 cells. However, the PYCR1 protein level in the OE+ mimic group was comparable to that in the blank group (Figure 5a). Next, the OE groups showed increased cell viability compared with the blank groups, while cells transfected with mimic+ OE counteracted this effect (Figure 5b). In addition, the OE groups showed approximately 1.5-fold upregulated cell proliferation compared with the blank groups, which was inhibited by the co-transfection with the mimic (Figure 5c). Moreover, the Bcl-2 protein in the OE group was significantly upregulated and Bax was downregulated compared with the blank group, and the effect was reversed by mimic+ OE (Figure 5d). Furthermore, the OE groups displayed a 1.3-fold increase in cell migration compared to the blank groups, whereas the role was repressed by cells transfected with mimic+ OE (Figure 6a). Finally, the OE groups showed a 1.3-fold increase in cell migration compared to the blank groups, but the mimic+ OE treatment suppressed this effect (Figure 6b). Collectively, these results showed that hsa-miR-150-5p attenuated the development of NPC by repressing PYCR1.

(a) Measurement of PYCR1 protein expression in C666-1 and SUNE-1 cells transfected with empty vector+mimic-NC, OE, mimic and mimic+ OE by western blot. (b) Cell viability was detected in C666-1 and SUNE-1 cells transfected with empty vector+mimic-NC, OE, mimic and mimic+ OE by CCK-8 assay. (c) Cell proliferation was detected in C666-1 and SUNE-1 cells transfected with empty vector+mimic-NC, OE, mimic and mimic+ OE by BrdU assay. (d) Bax and Bcl-2 protein expression was detected in C666-1 and SUNE-1 cells transfected with empty vector+mimic-NC, OE, mimic and mimic+ OE by western blot assay. n = 3. *, *P* < 0.05; **, *P* < 0.001 compared with blank. ^#^, *P* < 0.05; ^##^, *P* < 0.001 compared with mimic+ OE. NC, negative control; mimic, hsa-miR-150-5p mimic; OE, PYCR1 overexpression; mimic+OE, hsa-miR-150-5p mimic+ PYCR1 overexpression.

(a) Cell migration was detected in C666-1 and SUNE-1 cells transfected with empty vector+mimic-NC, OE, mimic, and mimic+ OE by BrdU assay. (b) Cell invasion was determined in C666-1 and SUNE-1 cells transfected with empty vector+mimic-NC, OE, mimic, and mimic+ OE. n = 3. *, *P* < 0.05; **, *P* < 0.001 compared with blank. ^#^, *P* < 0.05; ^##^, *P* < 0.001 compared with mimic+ OE. NC, negative control; mimic, hsa-miR-150-5p mimic; OE, PYCR1 overexpression; mimic+ OE, hsa-miR-150-5p mimic+ PYCR1 overexpression.

## Discussion

This study showed that hsa-miR-150-5p levels were downregulated in the NPC tissue samples and cells and were negatively correlated with the PYCR1 levels in the NPC tumor tissue samples. The upregulation of hsa-miR-150-5p inhibits NPC cell progression. Importantly, hsa-miR-150-5p suppressed the expression of *PYCR1*, which inhibited cell growth, migration, and invasion in NPC.

Over the years multiple studies have emphasized the biological role of hsa-miR-150-5p in carcinogenesis [30–32]. Zhou et al. reported that hsa-miR-150-5p was repressed by long non-coding RNA Myocardial Infarction Associated Transcript (lncRNA MIAT), and cell growth was increased by hsa-miR-150-5p mimics, and reversed by the overexpression of MIAT levels [30]. Tian et al. suggested that hsa-miR-150-5p significantly repressed the Wnt/β-catenin signaling pathway to hamper the stem cell-like characteristics of glioma cells [31]. Lou et al. reported that hsa-miR-150-5p was inhibited by lncRNA PART1, and the upregulation of hsa-miR-150-5p could prevent the oncogenesis of lncRNA PART1 by inhibiting *LRG1* expression [32]. Liu et al. found that hsa-miR-150-5p directly targeted the kinesin family member 3 C (*KIF3C*) gene to repress its expression and acts as a tumor suppressor in non-small cell lung cancer (NSCLC) progression [33]. Notably, Li et al. revealed that hsa-miR-150-5p reduced cell proliferation and tumorigenesis by arresting the G1/S phase transition in NPC cells [13]. Chan et al. suggested that the Wnt modulator ICG‑001 repressed cell growth by decreasing hsa-miR-150-5p levels and upregulating CD44 levels in NPC [11]. However, the upregulation of hsa-miR-150-5p promotes radioresistance of NPC cells by downregulating *GSK3β* expression [12]. Our study demonstrated that the hsa-miR-150-5p levels were dramatically downregulated in NPC cells. Furthermore, the upregulation of hsa-miR-150-5p attenuated the growth, migration, and invasion of NPC cells. Moreover, the overexpression of PYCR1 significantly inhibited the tumor suppressor effect of hsa-miR-150-5p on NPC.

Accumulating evidence has shown that PYCR1 exerts tumorigenic effects in a variety of cancers [27–29,34,35]. Sun et al. found that PYCR1 was upregulated by lncRNA TRPM2-AS, which sponges miR-140-3p, significantly promotes the growth of breast cancer cells [34]. Xiao et al. found that the overexpression of *PYCR1* significantly accelerated gastric cancer progression, and patients with high *PYCR1* expression had poor survival outcomes [29]. In addition, PYCR1 markedly facilitates the migration and invasion of NSCLC cells [35]. Furthermore, silencing of PYCR1 reduces the cell growth, drug resistance, and epithelial-mesenchymal transition (EMT) by inactivating the p38 mitogen-activated protein kinase (MAPK) and NF-κB signaling pathways in colorectal cancer cells [27]. The downregulation of PYCR1 dramatically repressed cell growth and survival by inactivating the c-Jun N-terminal kinase/insulin receptor substrate 1 (JNK/IRS1) pathway in hepatocellular cancer cells [28]. However, the role of PYCR1 in NPC development remains unclear. In our study, we demonstrated for the first time that *PYCR1* expression is conspicuously upregulated in NPC tissues and cells. The overexpression of *PYCR1* accelerated the growth, migration, and invasion of NPC cells Importantly, we found that hsa-miR-150-5p suppressed the effect of PYCR1, which further suppressed NPC cell progression.

As previously, evidences have revealed that PYCR1 dramatically enhanced cell growth and survival through activating JNK/IRS1 pathway in the development of hepatocellular cancer [28]. PYCR1 facilitated cell growth, drug resistance and EMT through activating p38 MAPK and NF-kappaB signaling pathways of colorectal cancer cells [27]. However, potential signaling pathways involved in hsa-miR-150-5p-PYCR1 axis in NPC needs further elucidation. In addition, the lack of prognostic analysis of NPC patients and in vivo experiments limit the persuasive power of this study to some extent. In the future, we will further explore the correlation between hsa-miR-150-5p-PYCR1 and patient survival and its effect on NPC tumor growth in vivo.

## Conclusions

This study revealed that hsa-miR-150-5p attenuated NPC tumorigenesis by reducing PYCR1 expression. Therefore, our study provides a comprehensive investigation of hsa-miR-150-5p and PYCR1 in NPC, which could provide a good potential therapeutic approach for treating patients with NPC.

## Supplementary Material

Supplemental Material

## Data Availability

The datasets used and analyzed during the current study are available from the corresponding author on reasonable request.

## List of abbreviations

miRNAs: MicroRNAs; NC: negative control; NSCC: non-small-cell lung cancer; OE: over expression; OSCC: oral squamous cell carcinoma; PYCR1: pyrroline-5-carboxylate reductase 1; UTRs: untranslated regions; WT: wild-type.
