# Supplementary material for: MicroRNA hsa-miR-150-5p inhibits nasopharyngeal carcinogenesis by suppressing PYCR1 (pyrroline-5-carboxylate reductase 1)
Source: Bioengineered. 2021 Dec 2;12(2):9766–78. doi: 10.1080/21655979.2021.1995102 (PMC8810012; doi:10.1080/21655979.2021.1995102)
Supplement: Supplemental Material [file KBIE_A_1995102_SM1378.zip › Supplementary Table 1.docx]

| Gene name | Primer type | Sequence |
| --- | --- | --- |
| miR-150-5p | forward | 5ʹ-TCGGCGTCTCCCAACCCTTGTAC-3ʹ |
|  | reverse | 5ʹ-GTCGTATCCAGTGCAGGGTCCGAGGT-3ʹ |
| CSE1L | forward | 5’-TGACCAA-CACTCCAGTCGTG-3’ |
|  | reverse | 5’-GTCCAGCTTCACCTTGTCCA-3’ |
| GAPDH | forward | 5’-GGAGCGAGATCCCTCCAAAAT-3’ |
|  | reverse | 5’-GGCT-GTTGTCATACTTCTCATGG-3’ |
| U6 | forward | 5ʹ-CTCGCTTCGGCAGCACATATACT-3ʹ |
|  | reverse | 5ʹ-ACGCTTCACGAATTTGCGTGTC-3ʹ |

Table 1. Sequence of PCR primers used in this study.
