# Supplementary material for: MicroRNA hsa-miR-150-5p inhibits nasopharyngeal carcinogenesis by suppressing PYCR1 (pyrroline-5-carboxylate reductase 1)
Source: Bioengineered. 2021 Dec 2;12(2):9766–78. doi: 10.1080/21655979.2021.1995102 (PMC8810012; doi:10.1080/21655979.2021.1995102)
Supplement: Supplemental Material [file KBIE_A_1995102_SM1378.zip › Supplementary Table 2.docx]

Supplementary table 2. The downregulated miRNAs in GSE118613

| miRNA_ID | adj.P.Val | logFC |
| --- | --- | --- |
| hsa-miR-451a | 7.98E-04 | -2.71 |
| hsa-let-7a-5p | 4.39E-04 | -2.59 |
| hsa-miR-16-5p | 1.14E-03 | -2.57 |
| hsa-miR-26a-5p | 1.53E-04 | -2.39 |
| hsa-miR-223-3p | 6.87E-04 | -2.32 |
| hsa-let-7f-5p | 7.98E-04 | -2.21 |
| hsa-miR-15b-5p | 3.03E-04 | -2.17 |
| hsa-miR-103a-3p | 2.43E-03 | -2.14 |
| hsa-let-7g-5p | 1.74E-03 | -2.08 |
| hsa-miR-4284 | 3.40E-04 | -2.06 |
| hsa-let-7i-5p | 3.13E-03 | -2.01 |
| hsa-miR-1273g-3p | 1.35E-02 | -1.89 |
| hsa-let-7d-5p | 4.74E-03 | -1.89 |
| hsa-miR-15a-5p | 1.15E-02 | -1.77 |
| hsa-miR-150-5p | 2.87E-03 | -1.76 |
| hsa-miR-155-5p | 2.54E-05 | -1.61 |
| hsa-miR-4774-3p | 2.98E-02 | -1.60 |
